# The News from the Committee Meeting

**Published:** 1885-09

**Authors:** 


### SUPPLEMENT.

Vol. 1.] AUSTIN, TEXAS, SEPTEMBER, 10, 1885. [No. 3.

### EDITORIAL.

### THE NEWS FROM THE COMMITTEE MEETING.

We are, indeed, the first to give the profession any report whatever of the action of the International Congress Committee, in New York on the 3d. After writing the closing paragraph of this issue, expressing disappointment in not getting a report, and after the journal was sent to the bindery, we received a letter from one of the physicians who was present, giving us the following items, which we hasten to lay before our readers, knowing the feverish anxiety on the subject:

First and most glorious of all,—our own—the noble old Roman, N. S. Davis, was made SECRETARY GENERAL of the Congress!! has accepted and gone actively to work!! The attendance was as large as at Chicago,—and the meeting was harmonious, twenty-seven committee-men being present, representing the whole *extent* of the American Union in length and breadth, including COLE, of California, (the President of the Committee;) Battey, of Georgia; Sim, of Tennessee; Wathen, of Kentucky; Gordon, of Maine; and ———, of Nebraska: (literally "from Maine to California.") The qualifications for membership in the Congress were made more liberal; and *thirty days grace were given those who had resigned, to come back*,—except Chairmen of Sections, who are out to stay. All the vacant chairman-ships were filled. Amongst the new chairmen are W. H. Pancoast, Anatomy; A. W. Calhoun, of Georgia, Ophthalmology; — Robinson (A. R. or Beverly?) on Dermatology; Briggs, of Nashville, on Surgery (vice Yandell, "declined"); and Edmund S. F. Arnold, of New York, was made Treasurer of the Congress.

The Association Journal of the 12th will contain the complete list, and further particulars; meantime we are enabled to give our readers the above, which is enough to relieve the suspense, and to assure them of the success of our cause.

We might make several apt quotations—such as "Truth crushed to earth," you know, but space is space just now, and we desist. It will be observed that the South has come in for more honors. If one does not stand up for his rights in this world, he will very often not get those rights. Suppose the South had not said anything against the appointments as first proposed? She would have been entirely slighted. We opine the indignation will be great amongst European physicians when they come to realize, which they must do shortly, that they have been "played for suckers," and made to believe there was no respectable medical talent in America outside of twenty-eight Philadelphians. A Congress with Flint, Davis, Pancoast, Campbell, and Batty and others whose names are of world-wide familiarity, can be nothing short of a grand success;—at least we will "stand pat" on that hand. We will here record the prediction, however, that not one of those who have bitten off their noses, will come back. Well, then, let them go. Shake, Brother Shoemaker.

### DROPPED FROM THE ROLL.

The efficient and courteous Secretary of the Texas State Medical Association, Dr. W. J. Burt—(by the by, the Doctor is now down and suffering with the prevailing fever—dengue; he and his young son, Eugene, both being stricken down the same day) informs us that about sixty names of members of the State Association have been dropped from the roll for non payment of dues. Those who do not receive a copy of the Transactions will thus know why. Dr. Burt requests us to say, however, under the Constitution and By-Laws, those members can be reinstated upon the payment of arrears.

Volume 1, number 1, of DANIEL'S MEDICAL JOURNAL, is at hand. It is edited and published by Dr. F. E. Daniel, of Austin, Texas, and late of the *Texas Courier-Record of Medicine.* To say that it is a beauty is putting it mildly: it is a daisy--a jewel. We never saw a more handsomely gotten up periodical than this. Doctor Daniel's salutatory is an exceedingly fine literary effort. There can be no doubt that the Doctor's soul is in the work, and that it will "march on" to success. The JOURNAL is in a high degree sprightly, interesting and practical. Subscription price, $2 per annum; single copies, 25 cents.—*Medcal World.*

